# Multi-Acting Mitochondria-Targeted Platinum(IV) Prodrugs of Kiteplatin with α-Lipoic Acid in the Axial Positions

**DOI:** 10.3390/ijms19072050

**Published:** 2018-07-14

**Authors:** Salvatore Savino, Cristina Marzano, Valentina Gandin, James D. Hoeschele, Giovanni Natile, Nicola Margiotta

**Affiliations:** 1 Department of Chemistry, University of Bari Aldo Moro, Via E. Orabona 4, 70125 Bari, Italy; salvatoresavino.s@libero.it; 2 Department of Pharmaceutical and Pharmacological Sciences, University of Padova, Via Marzolo 5, 35131 Padova, Italy; cristina.marzano@unipd.it (C.M.); valentina.gandin@unipd.it (V.G.); 3 Department of Chemistry, Eastern Michigan University, Ypsilanti, MI 48197, USA; hoeschel@chemistry.msu.edu

**Keywords:** platinum(IV) complexes, cisplatin, kiteplatin, α-lipoic acid, DNA, mitochondria, tumor spheroids

## Abstract

Platinum(II) drugs are activated intracellularly by aquation of the leaving groups and then bind to DNA, forming DNA adducts capable to activate various signal-transduction pathways. Mostly explored in recent years are Pt(IV) complexes which allow the presence of two additional ligands in the axial positions suitable for the attachment of other cancer-targeting ligands. Here we have extended this strategy by coordinating in the axial positions of kiteplatin ([PtCl_2_(*cis*-1,4-DACH)], DACH = Diaminocyclohexane) and its CBDCA (1,1-cyclobutanedicarboxylate) analogue the antioxidant α-Lipoic acid (ALA), an inhibitor of the mitochondrial pyruvate dehydrogenase kinase (PDK). The new compounds (*cis*,*trans*,*cis*-[Pt(CBDCA)(ALA)_2_(*cis*-1,4-DACH)], **2**, and *cis*,*trans*,*cis*-[PtCl_2_(ALA)_2_(*cis*-1,4-DACH)], **3**), after intracellular reduction, release the precursor Pt(II) species and two molecules of ALA. The Pt residue is able to target DNA, while ALA could act on mitochondria as activator of the pyruvate dehydrogenase complex, thus suppressing anaerobic glycolysis. Compounds **2** and **3** were tested in vitro on a panel of five human cancer cell lines and compared to cisplatin, oxaliplatin, and kiteplatin. They proved to be much more effective than the reference compounds, with complex **3** most effective in 3D spheroid tumor cultures. Notably, treatment of human A431 carcinoma cells with **2** and **3** did not determine increase of cellular ROS (usually correlated to inhibition of mitochondrial PDK) and did not induce a significant depolarization of the mitochondrial membrane or alteration of other morphological mitochondrial parameters.

## 1. Introduction

**Scheme 1:**
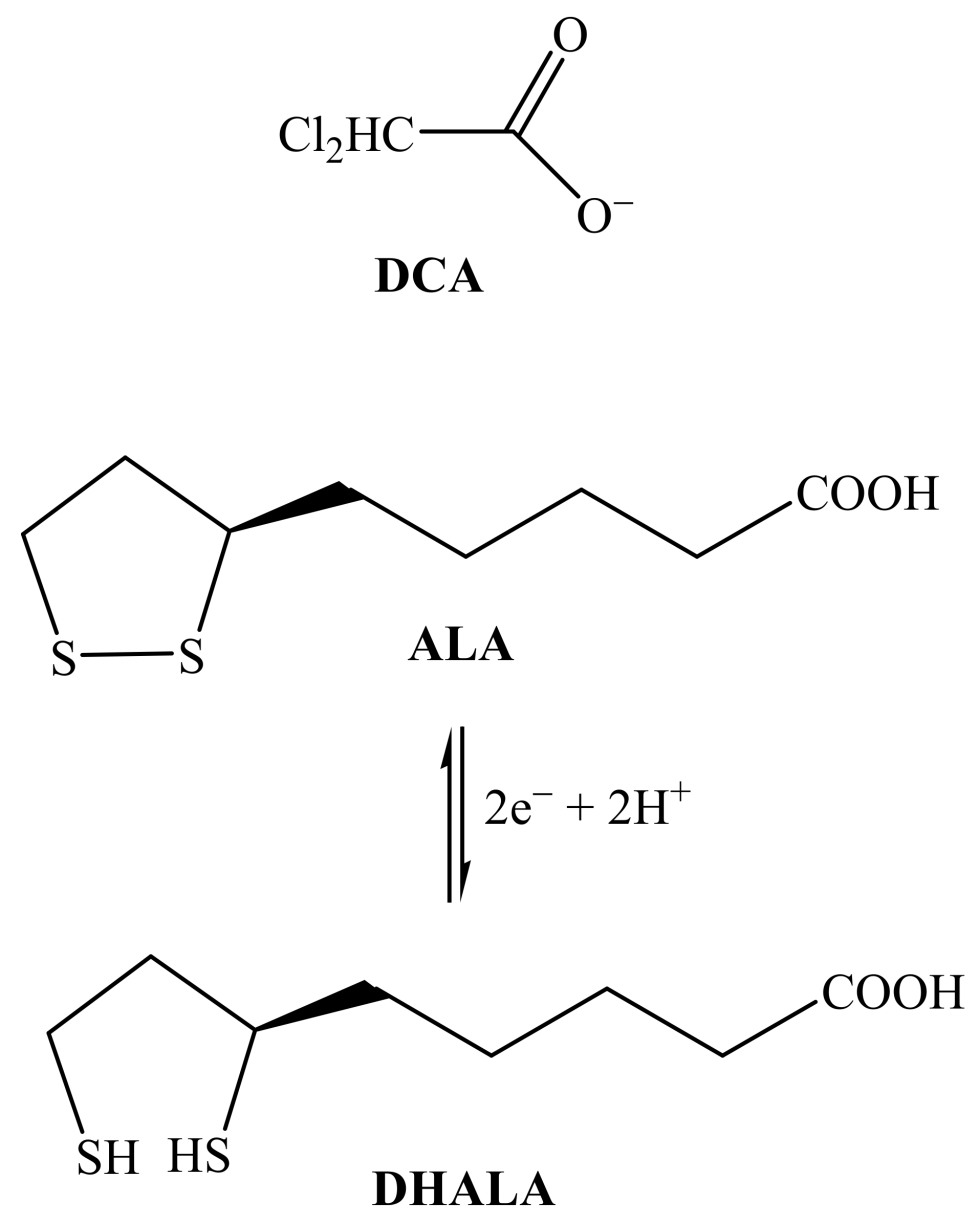
Molecular structures of dichloroacetate (DCA), α-lipoic acid (ALA), and α-dihydrolipoic acid (DHALA).

It is well known that numerous cancer cells preferentially convert glucose to lactate, even in the presence of oxygen (aerobic glycolysis), for the generation of ATP. This phenomenon was discovered by Warburg already in the 1920s and is hence reported as the Warburg effect [1,2]. Yet, aerobic glycolysis is not very efficient from an energetical point of view since it leads to the production of only two molecules of ATP per molecule of glucose while the complete oxidation of glucose to carbon dioxide and water produces 32 molecules of ATP. For this reason, cancer cells need huge amounts of glucose to satisfy their high demand of energy, but, at the same time, they are able to adapt to hypoxic conditions. Cancer therapy can take advantage of the key feature of glucose metabolism of cancer cells. The pyruvate dehydrogenase complex is located in the mitochondrial matrix of eukaryotes; the complex acts to convert pyruvate (a product of glycolysis in the cytosol) to acetyl-coA, which is then oxidized in the mitochondria to produce energy in the citric acid cycle. Pyruvate dehydrogenase kinase (PDK) is a kinase enzyme which acts to inactivate the pyruvate dehydrogenase enzyme by phosphorylating it using ATP; by downregulating the pyruvate dehydrogenase, PDK will decrease the oxidation of pyruvate in mitochondria and increase the conversion of pyruvate to lactate in the cytosol. An inhibitor of PDK will activate those enzymes that are able to shift the metabolism toward the complete oxidation of glucose. A compound that has been used for such purpose (inhibition of PDK) is dichloroacetate (DCA; Scheme 1), an orphan drug. DCA works to counteract the increased production of lactate exhibited by tumor cells (anaerobic respiration) by activating the pathway to pull the intermediates into the citric acid cycle and finish off with oxidative phosphorylation (aerobic respiration) [3].

Also the dithiol compound (*R*)-(+)-α-lipoic acid (6,8-dithio-octanoic acid; ALA, Scheme 1) is a potential activator of the pyruvate dehydrogenase complex and can be potentially used to suppress anaerobic glycolysis [4,5]. ALA is synthesized from octanoic acid in the mitochondria and it is also taken from food. Apart from being capable to chelate metal ions, ALA has a unique reductive power and its disulfide group can be easily reduced to form α-dihydrolipoic acid (DHALA; Scheme 1) and both constitute a low redox potential pair (E’_0_ = −0.29 V) [6] capable to scavenge a variety of reactive oxygen species (ROS) [7,8,9,10].

ROS and, in general, oxidative stress also play a crucial role in cancer cells being correlated with cell growth and apoptosis [11]. In particular, ALA was shown to activate apoptosis in human cancer cell lines by inducing a reversible cell-cycle arrest but failed to trigger apoptosis in non-transformed cell lines [12]. Apoptosis was potentiated by ALA also in human leukemia cells and, because of its antioxidant properties, it was suggested that this compound can promote a reducing intracellular environment that is necessary for the activation of caspases [13]. On the other hand, in neurons and in hepatocytes [14,15], ALA was demonstrated to exert a protective activity against apoptosis, thus showing an opposite activity in tumor and healthy cells.

In another study, exposure of HT-29 human colon cancer cells to ALA caused a dose-dependent increase of caspase-3-like activity associated with DNA fragmentation. Moreover, in this tumor cell line ALA was not able to scavenge cytosolic O_2_^·−^ whereas it was capable to increase the generation of the superoxide anion radical in the mitochondria [16] preceded by an increased influx of lactate or pyruvate into the organelles. Oppositely to HT-29 colon cancer cells, no apoptosis was observed in nontransformed human colonocytes, thus providing evidences for a selective induction of apoptosis in the cancer cell line by a prooxidant mechanism initiated by an increased uptake of oxidizable metabolites in the mitochondria.

In vitro cell proliferation inhibition by ALA was also reported for neuroblastoma cell lines Kelly, SK-N-SH, and Neuro-2a, for breast cancer cell lines SkBr3 [16] and MDA-MB-231, as well as for leukemia cells Jurkat and CCRF-CEM [17,18]. Moreover, in a xenograft mouse model with subcutaneous SkBr3 cells, daily treatment with ALA significantly retarded tumor growth, further supporting the potential anticancer activity of this compound.

**Scheme 2:**
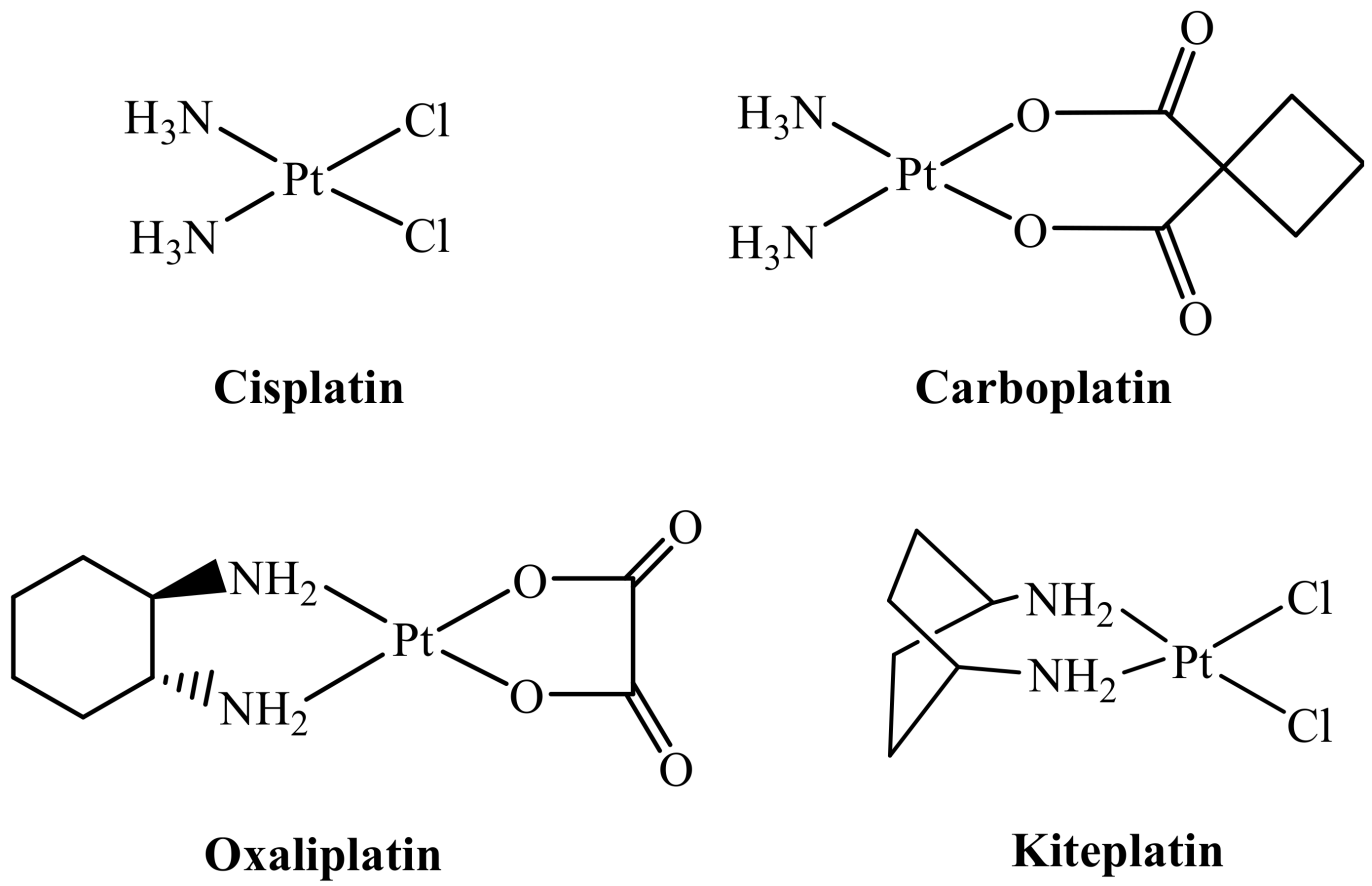
Sketches of clinically used platinum(II) drugs (Cisplatin, Carboplatin, and Oxaliplatin) and of Kiteplatin.

Platinum(II) complexes are well-known antiproliferative agents [19] and, among them, cisplatin, carboplatin, and oxaliplatin (Scheme 2) have received Food and Drug Administration approval and are used in the clinic worldwide [19,20,21]. However, the appearance of resistance and induction of severe side effects limit the use of these drugs [20]. In order to overcome at least some of these drawbacks, new platinum drugs based on the platinum(IV) core have been developed. In this context, we have recently focused on Pt(IV) complexes [22,23] derived from kiteplatin, [PtCl_2_(*cis*-1,4-DACH)] (DACH = diaminocyclohexane; Scheme 2) including a derivative having two DCA ligands in axial positions [24]; the aim was to obtain a dual acting complex endowed with the ability to target both nuclear DNA and mitochondria. Kiteplatin contains an isomeric form of the diamine ligand 1*R*,2*R*-DACH present in oxaliplatin and is effective against both cisplatin-resistant (ovarian C13*) and oxaliplatin-resistant (colon LoVo-OXP) cancer cell lines, suggesting that the spectrum of activity of this compound could be different from that of cisplatin and oxaliplatin [25,26,27,28]. The newly synthesized Pt(IV) complexes with DCA, *cis*,*trans*,*cis*-[PtCl_2_(DCA)_2_(*cis*-1,4-DACH)] and *cis*,*trans*,*cis*-[Pt(CBDCA)(DCA)_2_(*cis*-1,4-DACH)] (CBDCA = 1,1-cyclobutanedicarboxylate), were tested in vitro against a series of different tumor cell lines, some of which were selected for their resistance to cisplatin and oxaliplatin, and the antitumor activity of the lead compound, *cis*,*trans*,*cis*-[PtCl_2_(DCA)_2_(*cis*-1,4-DACH)], was also assessed in vivo in a syngeneic murine model of solid tumor (the Lewis Lung Carcinoma).

Tested compounds induced a substantial increase of ROS production, blockage of oxidative phosphorylation, hypopolarization of the mitochondrial membrane, and caspase-3/7-mediated apoptotic cell death. These effects could be a consequence of the DCA released after intracellular reduction of the Pt(IV) complexes.

**Scheme 3:**
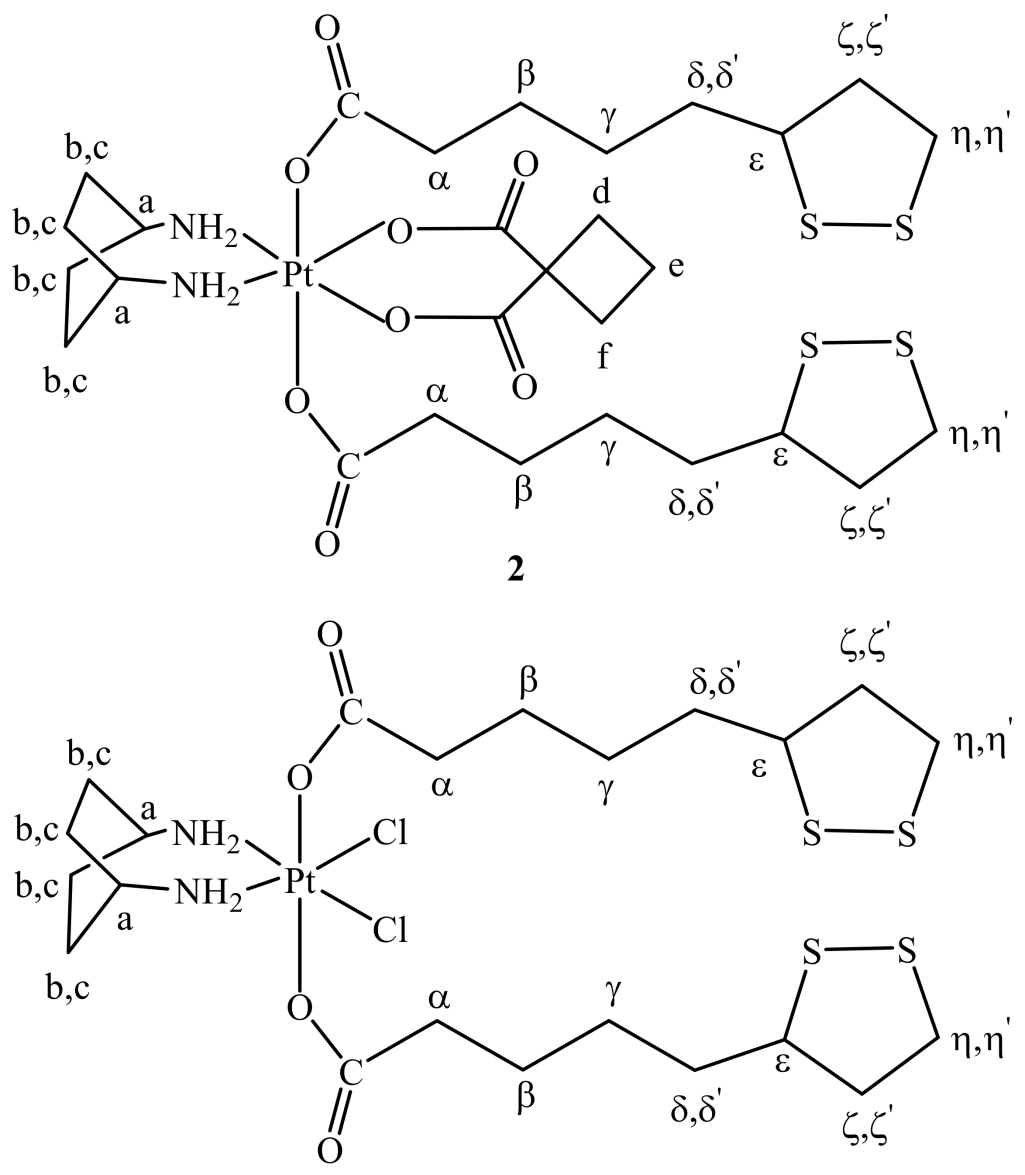
Sketches of the two novel kiteplatin Pt(IV) derivatives investigated in this work: *cis*,*trans*,*cis*-[Pt(CBDCA)(ALA)_2_(*cis*-1,4-DACH)] (**2**) and *cis*,*trans*,*cis*-[PtCl_2_(ALA)_2_(*cis*-1,4-DACH)] (**3**).

On this basis we decided to explore the conjugation of ALA (another activator of the pyruvate dehydrogenase complex like DCA) in the axial positions of a Pt(IV) derivative of kiteplatin and in this paper we report the synthesis, characterization, and biological activity of two kiteplatin derivatives, *cis*,*trans*,*cis*-[Pt(CBDCA)(ALA)_2_(*cis*-1,4-DACH)] (**2**; Scheme 3) and *cis*,*trans*,*cis*-[PtCl_2_(ALA)_2_(*cis*-1,4-DACH)] (**3**; Scheme 3).

It could be expected that the new compounds could mitigate some side effects of platinum-drug therapy such as ototoxicity and nephrotoxicity. In fact, it has been shown that pretreatment with ALA significantly reduces apoptotic cell death of the inner and outer hair cells in cisplatin-treated organ of Corti explants and attenuates ototoxicity via marked lowering of the expression levels of proinflammatory cytokines and other cisplatin-induced biomarkers of ototoxicity in cisplatin-treated HEI-OC1 cells [29]. Moreover, it has been shown that lipoic acid also prevents cisplatin-induced nephrotoxicity in rats [30].

## 2. Results and Discussion

### 2.1. Synthesis and Characterization

It was demonstrated that compounds that support pyruvate dehydrogenase reaction (such as DCA and ALA) are promising agents in cancer therapy since they are able to target the glucose metabolism of cancer cells [31]. In particular, the antioxidant ALA is able to induce apoptosis in HT-29 human colon cancer cells via an increased ROS production in mitochondria by enhancing the uptake of pyruvate and lactate from glycolysis into the mitochondria [16]. Oxidation of the monocarboxylates pyruvate and lactate in the citric acid cycle increases the delivery of reductive equivalents to the respiratory chain with the final result of drastically increasing the mitochondrial production of O_2_^·−^ which, in turn, triggers apoptosis in the cancer cells.

We have already reported on kiteplatin-Pt(IV) complexes having DCA in the axial positions with the lead compound, *cis*,*trans*,*cis*-[PtCl_2_(DCA)_2_(*cis*-1,4-DACH)] showing encouraging antitumor activity also in vivo in the solid syngeneic murine model Lewis Lung Carcinoma [24]. In this work we have extended the investigation to Pt(IV) kiteplatin derivatives containing, in the axial positions, the antioxidant ALA: namely *cis*,*trans*,*cis*-[Pt(CBDCA)(ALA)_2_(*cis*-1,4-DACH)] (**2**) and *cis*,*trans*,*cis*-[PtCl_2_(ALA)_2_(*cis*-1,4-DACH)] (**3**).

Lipoic acid has been already used as a ligand in platinum complexes. In particular, an amide formed by lipoic acid and aniline was used as a linker between a platinum complex and gold nanoparticles [32], while polynuclear platinum complexes with lipoic acid were patented for the prophylaxis or treatment of cancer [33]. To the best of our knowledge, this is the first time that mononuclear platinum complexes with lipoic acid have been prepared and tested in vitro for their potential application as antitumor drugs.

Naturally occurring α-Lipoic acid is in the (*R*)-(+) configuration, however, in this work we have used the commercially available racemic (±)-α-Lipoic acid that was activated into its anhydride by DCC conjugation and then reacted with the Pt(IV)-dihydroxido derivative of kiteplatin *cis*,*trans*,*cis*-[Pt(CBDCA)(OH)_2_(*cis*-1,4-DACH)] (**1**). The NMR characterization of compound **1** is reported in the Experimental Section and in the Supplementary Information (Figures S1–S3).

**Figure 1:**
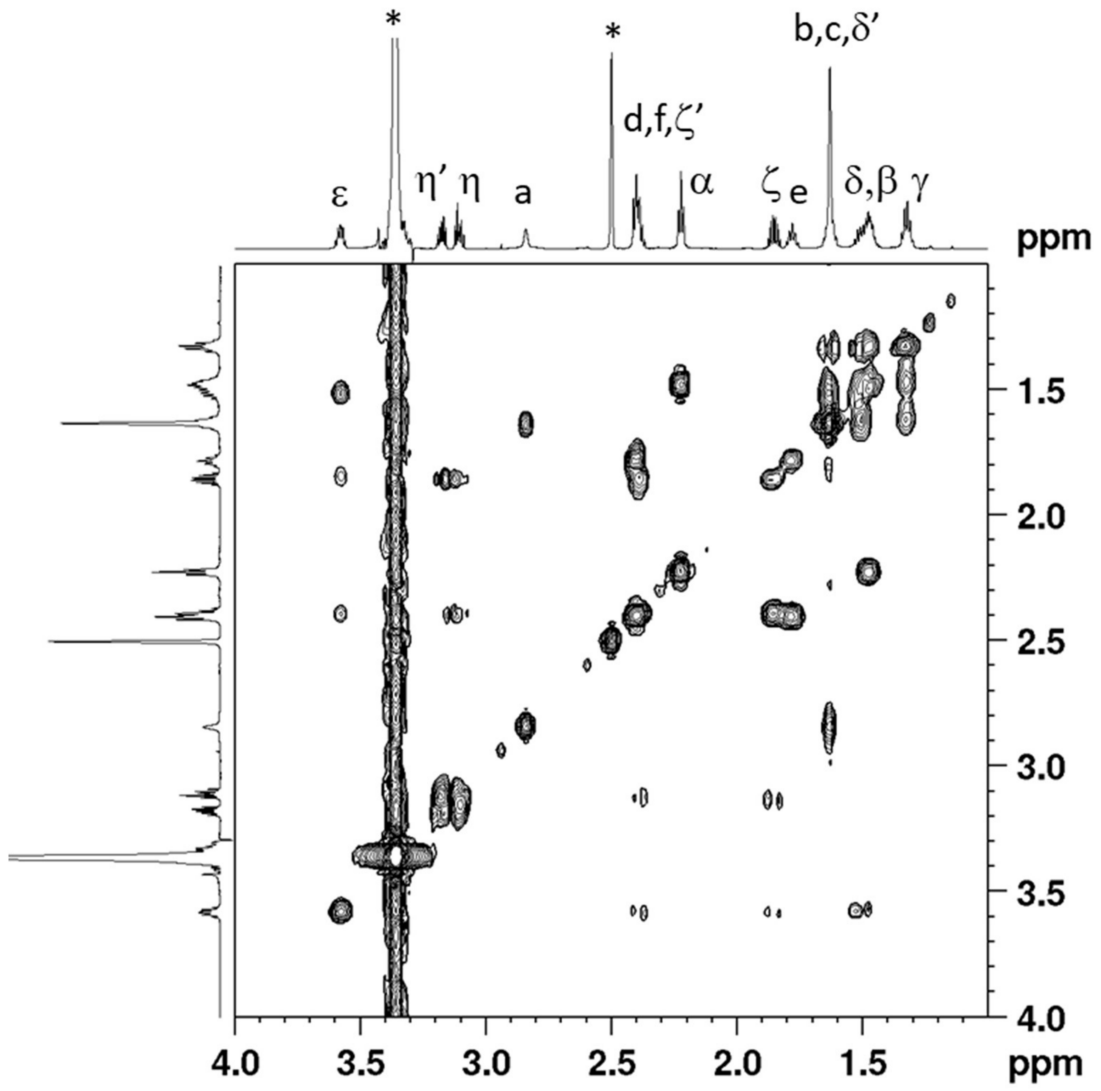
Selected region of the 2D COSY (700 MHz) spectrum obtained for *cis*,*trans*,*cis*-[Pt(CBDCA)(ALA)_2_(*cis*-1,4-DACH)] (**2**) in DMSO-d_6_. Numbering of protons is reported in Scheme 3. The asterisks indicate residual solvent peaks.

*Cis*,*trans*,*cis*-[Pt(CBDCA)(ALA)_2_(*cis*-1,4-DACH)] (**2**) has been characterized by elemental analysis, ESI-MS, and NMR. The ESI-MS (+) spectrum showed the presence of a peak at *m*/*z* = 885.15 corresponding to [**2** + Na]^+^ and the experimental isotopic pattern was in good agreement with the theoretical one (data not shown). The NMR characterization of compound **2** was obtained with the help of a 2D COSY experiment (Figure 1). Assignment of protons started from the methylenic protons of coordinated ALA. The triplet integrating for 4 protons falling at 2.22 ppm and the multiplet at 1.48 ppm, correlated by a COSY cross peak, were assigned to CH_2_ in α and β positions, respectively, of the lipoato ligand (see Scheme 3 for numbering of protons). The multiplet located at 1.32 ppm was assigned to the CH_2_γ protons since it shows COSY cross peaks with the CH_2_ in β position and the multiplets at 1.62 and 1.51 ppm; these latter were assigned to the protons in *δ*’ and *δ* positions, respectively. The 1,2-dithiolane ring showed 5 different signals. The multiplet resonating at 3.58 ppm was assigned to the *ε* CH based on a COSY a cross peak with the proton *δ* and two additional cross peaks with the multiplets resonating at 2.39 and 1.85 ppm assigned, respectively, to the methylenic protons ζ’ and ζ. The characterization of the lipoato ligand ends with the attributions of the multiplets at 3.17 and 3.11 ppm to the methylenic protons *η*’ and *η*, these latter signals showing cross peaks with the protons ζ and ζ’.

**Figure 2:**
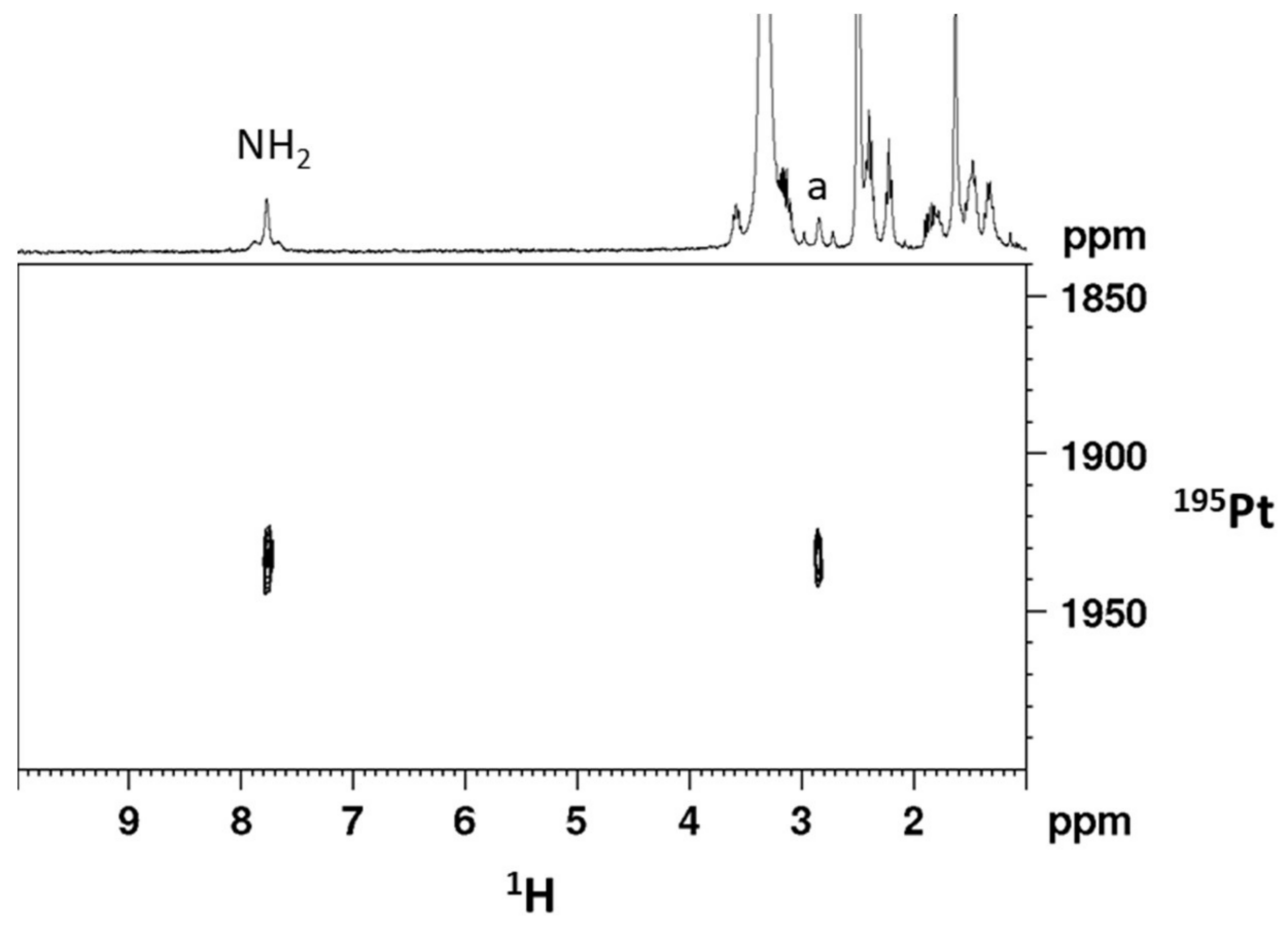
[^1^H, ^195^Pt]-HSQC 2D spectrum (^1^H 300 MHz) obtained for *cis*,*trans*,*cis*-[Pt(CBDCA)(ALA)_2_(*cis*-1,4-DACH)] (**2**) in DMSO-d_6_.

With reference to the *cis*-1,4-DACH ligand, the singlet with platinum satellites (^2^*J*_H-Pt_ = 66 Hz; see Figure 2) located at 7.76 ppm was assigned to the aminic protons, at lower field with respect to the corresponding signal in compound **1** (6.47 ppm). The deshielding is probably due to the presence of the carboxylic groups of coordinated ALA in axial positions (hydrogen bonds between axial C=O and coordinated NH_2_ were found in similar systems) [34]. The methynic (H_a_) and methylenic (H_b,c_) protons of coordinated DACH give, respectively, a singlet at 2.84 and a broad signal (integrating for eight protons) at 1.63 ppm. Finally, the multiplet at 2.40 ppm (partially overlapping with ζ’) and the quintet at 1.78 ppm were assigned to the cyclobutane protons of CBDCA.

The [^1^H-^195^Pt]-HSQC 2D NMR spectrum of compound **2** in DMSO-d_6_ (Figure 2) exhibits two cross peaks, located at 7.76/1932.7 and 2.84/1932.7 ppm (^1^H/^195^Pt), correlating the aminic and methynic protons of *cis*-1,4-DACH with the platinum atom. The ^195^Pt chemical shift is found at lower field with respect to that of the precursor compound **1** (1617.6 ppm in DMSO-d_6_) but is in good agreement with that reported for similar Pt(IV) dicarboxylato derivatives with the platinum atom in a N_2_O_4_ coordination environment [35].

**Figure 3:**
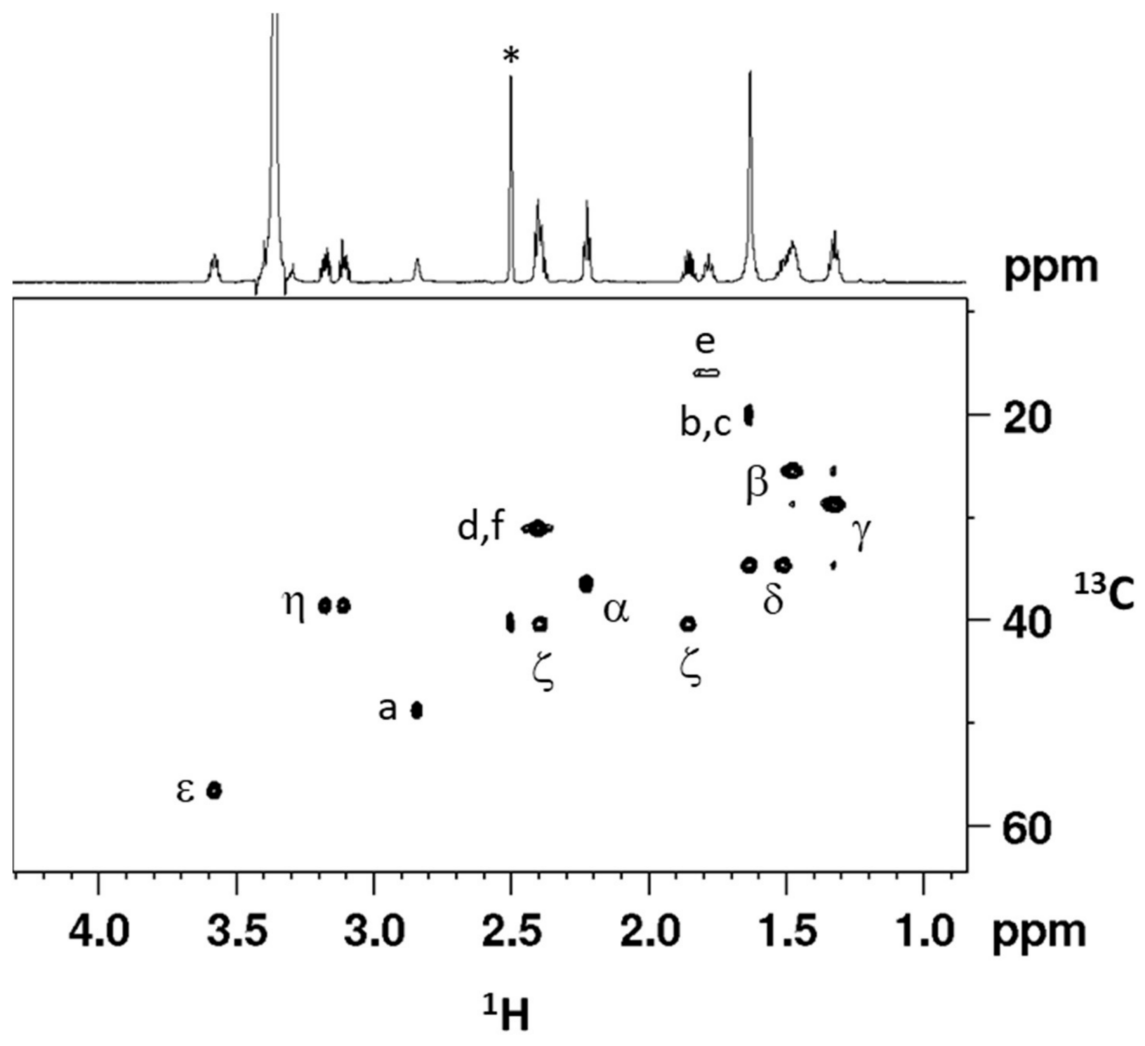
[^1^H, ^13^C]-HSQC (^1^H 700 MHz) of *cis*,*trans*,*cis*-[Pt(CBDCA)(ALA)_2_(*cis*-1,4-DACH)] (**2**) in DMSO-d_6_. Numbering of protons is reported in Scheme 3. The asterisk indicates the residual solvent peak.

The assignment of ^13^C signals has been accomplished by a [^1^H, ^13^C]-HSQC 2D NMR spectrum (Figure 3) and data are reported in the Experimental Section.

Compound **3**, *cis*,*trans*,*cis-*[PtCl_2_(ALA)_2_(*cis*-1,4-DACH)], was prepared with a procedure similar to that used for compound **2**, the only differences being the starting platinum(IV) derivative of kiteplatin, *cis*,*trans*,*cis-*[PtCl_2_(OH)_2_(*cis*-1,4-DACH)], and the purification step that required the use of a less polar solvent such as *n*-pentane. Compound **3** has been characterized by elemental analysis, ESI-MS, and NMR. The ESI-MS (+) spectrum showed the presence of a peak at *m*/*z* = 813.07 corresponding to [**3** + Na]^+^ and the experimental isotopic pattern of the peak resulted to be in agreement with the theoretical one (data not shown). The NMR characterization (in DMSO-d_6_) of compound **3** was obtained with the help of a 2D COSY experiment (Figure S4) and is similar to that reported for complex **2** with the exclusion of the signals belonging to the CBDCA ligand. The assignment of ^13^C signals has been accomplished by a [^1^H, ^13^C]-HSQC 2D NMR spectrum (Figure S5). The [^1^H-^195^Pt]-HSQC 2D NMR spectrum of compound **3** in DMSO-d6 (Figure S6) exhibits two cross peaks, located at 8.19/1217.6 and 2.98/1217.6 ppm (^1^H/^195^Pt), correlating the aminic and methynic protons of *cis*-1,4-DACH with the platinum atom. The ^195^Pt chemical shift is found at lower field with respect to that of the precursor compound *cis*,*trans*,*cis-*[PtCl_2_(OH)_2_(*cis*-1,4-DACH)] (964.6 ppm in DMSO-d_6_) [36] and is in good agreement with that reported for similar Pt(IV) derivatives with the platinum atom in a Cl_2_N_2_O_2_ coordination environment [37,38]. All the NMR data are reported in the Experimental Section while the NMR spectra are reported in the Supplementary Information (Figures S4–S6).

### 2.2. Biological Assays

The in vitro antitumor potency of the ALA Pt(IV) kiteplatin-derivatives **2** and **3** was evaluated on a panel of human cancer cells and compared to that of cisplatin (CDDP), oxaliplatin (OXP), and kiteplatin as well as to that of [Pt(CBDCA)(*cis*-1,4-DACH)]. Cell lines representative of lung (H157), colon (HCT-15), breast (MCF-7), cervical (A431), and ovarian (2008) carcinoma have been included. The cytotoxicity was evaluated by means of the MTT test for 72 h incubation with different concentrations of the tested compounds. IC_50_ values, calculated from dose-survival curves, are reported in Table 1.

The two ALA derivatives **2** and **3** proved to be much more effective than the reference compounds kiteplatin and [Pt(CBDCA)(*cis*-1,4-DACH)]. The higher potency of complex **3**, compared to that of complex **2**, reflects the one order magnitude higher potency of kiteplatin compared to that of [Pt(CBDCA)(*cis*-1,4-DACH)]. However, an effect due to the release of ALA after reduction of the Pt(IV) complexes is also evident. Notably, over the five tested cell lines the IC_50_ values of **2** and **3** were, in the order, about 7 and 4 times lower than those of the reference Pt(II) complexes. In addition, both Pt(IV) complexes showed, on average, an in vitro antitumor potency superior to those of the reference metallodrugs CDDP and OXP. Among Pt(IV) ALA derivatives, complex **3** was the most effective with an in vitro antitumor potential roughly an order of magnitude higher than that of CDDP and about 5.5 times greater than that of OXP.

The marked cell-killing effect observed against human A431 squamous cervical carcinoma cells prompted us to evaluate the in vitro antitumor activity of the Pt(IV) ALA derivatives on 3D cell cultures. As opposed to 2D monolayer cultures, cells growing in 3D culture systems form spheroids that are comprised of cells in various stages. The outer layers of the spheroid, being highly exposed to the medium, are mainly comprised of viable, proliferating cells whereas the core cells receiving less oxygen, growth factors, and nutrients, tend to be in a quiescent or hypoxic state [39]. Such cellular heterogeneity resembles that of in vivo tumors, making 3D cell cultures more predictive than conventional 2D monolayer cultures in screening antitumor drugs. Table 2 summarizes the IC_50_ values obtained after treatment of 3D cell spheroids of human A431 cervical cancer cells with the Pt(IV) ALA complexes as well as kiteplatin, [Pt(CBDCA)(*cis*-1,4-DACH)], CDDP, and OXP used as references.

Consistently with 2D studies, complex **3** proved to be the most effective compound, showing an efficacy (in decreasing cancer spheroid viability) about 2 times higher than those of CDDP, OXP, and kiteplatin. Conversely, complex **2** was slightly more effective than CDDP, OXP, and kiteplatin but markedly less cytotoxic than **3**.

Based on previous findings highlighting the ability of ALA derivatives to affect PDK (thus leading to ROS production and mitochondria hampering) [16,17,18], we investigated the effects of Pt(IV) ALA kiteplatin derivatives on mitochondria. In particular, we investigated the ROS production and the alteration of the mitochondrial membrane potential and of the mitochondrial morphological parameters. A preliminary NMR investigation revealed that the ALA ligands conjugated in the axial positions of the Pt(IV) complexes maintained their oxidized state also in the presence of glutathione. This finding was in line with literature data reporting the potentials of redox systems such as NAD+/NADH, GSSG/GSH, dehydroascorbate/ascorbate, etc. [6]. Hence, we are confident that the ALA ligand is released after the complexes have entered the tumor cells and can be reduced only after reduction of the Pt(IV) complexes to their Pt(II) counterparts.

**Figure 4:**
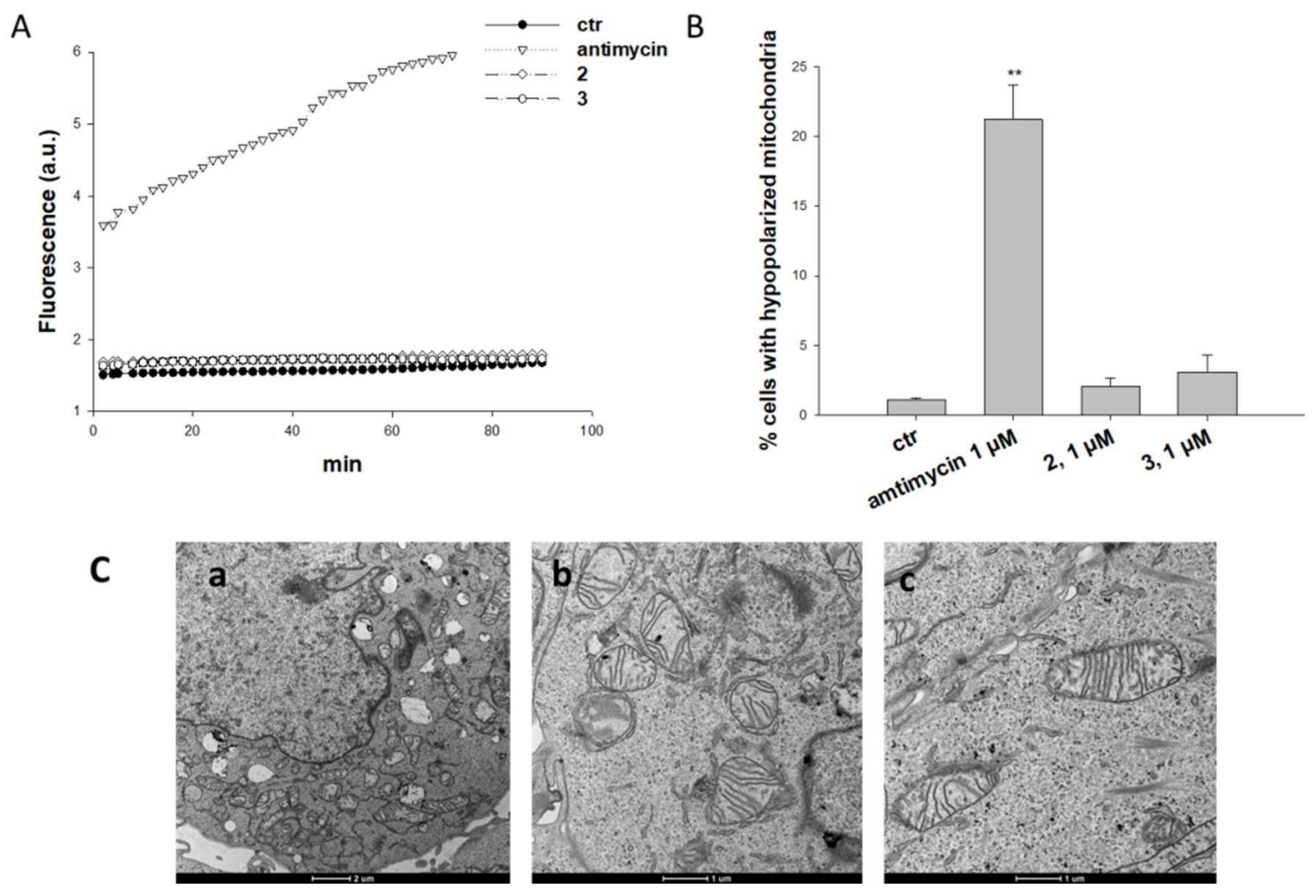
Effects induced by Pt(IV) ALA kiteplatin derivatives at mitochondrial level. (**A**) ROS production in A431 cells. Cells were pre-incubated in PBS/10 mM glucose medium for 20 min at 37 °C in the presence of 10 mM CM-H_2_DCFDA and then treated with **2**, **3**, or antimycin (1 μM). Fluorescence of DCF was measured at 485 nm (excitation) and 527 nm (emission). (**B**) Effects on mitochondrial membrane potential. A431 cells were treated for 24 h with **2**, **3**, or antimycin (1 μM) and stained with TMRM (10 nM). Fluorescence was estimated at 490 nm (excitation) and 590 nm (emission). Data are the means of five independent experiments. Error bars indicate S.D. ** *p* < 0.01. (**C**) TEM analysis of A431 cells treated for 24 h with 1 µM of **2** and **3**. Cells were processed through standard procedures as reported in the Experimental section. (**a**) Control; (**b**) **2**; (**c**) **3**.

Treatment of A431 cells with derivatives **2** and **3** did not determine any substantial increase in cellular ROS basal production (Figure 4A), whereas 2 h treatment with antimycin, a classical inhibitor of the mitochondrial respiratory chain at the level of complex III, caused a remarkable increase of the hydrogen peroxide content (about 6 times greater than that of control cells). Consistently, treatment with **2** and **3** did not induce a significant increase of A431 human cancer cells with depolarized mitochondria (Figure 4B) or any morphological alteration of mitochondria parameters (Figure 4C). Indeed, mitochondria of A431 cancer cells treated with **3** were conserved in shape and ultrastructure (cristae).

Overall, these results suggest that **2** and **3** do not activate a macroscopic antimitochondrial mechanism.

## 3. Materials and Methods

### 3.1. Chemicals, Instrumentation, and Platinum Complexes Precursors

Commercial reagent grade chemicals ((±)-α-Lipoic acid, dicyclohexylcarbodiimmide (DCC), 4-(dimethylamino)pyridine; Sigma-Aldrich, Milan, Italy) and solvents were used as received without further purification. ^1^H-NMR, COSY, and [^1^H, ^13^C]-HSQC 2D NMR spectra were recorded on Bruker Avance DPX 300 MHz and Bruker Avance III 700 MHz instruments. ^1^H and ^13^C chemical shifts were referenced using the internal residual peak of the solvent (DMSO-d_6_: 2.50 ppm for ^1^H and 39.51 ppm for ^13^C). [^1^H, ^195^Pt]-HSQC spectra were recorded on Bruker Avance DPX 300 MHz instrument (Bruker Italia S.r.L., Milano, Italy). ^195^Pt NMR spectra were referenced to K_2_PtCl_4_ (external standard placed at −1620 ppm with respect to Na_2_[PtCl_6_]) [37]. Electrospray Mass Spectrometry: ESI-MS was performed with a dual electrospray interface and a quadrupole time-of-flight mass spectrometer (Agilent 6530 Series Accurate-Mass Quadrupole Time-of- Flight (Q-TOF) LC/MS; Agilent Technologies Italia S.p.A., Cernusco sul Naviglio, Italy). Elemental analyses were carried out with an Eurovector EA 3000 CHN Instrument (Eurovector S.p.A., Milano, Italy).

Kiteplatin, [Pt(CBDCA)(*cis*-1,4-DACH)] (DACH = diaminocyclohexane; CBDCA = 1,1-cyclobutanedicarboxylate) [40] and *cis*,*trans*,*cis*-[PtCl_2_(OH)_2_(*cis*-1,4-DACH)] [36] were prepared according to already reported procedures and all analytical data were in good agreement with the given formulation.

### 3.2. Synthesis of cis,trans,cis-[Pt(CBDCA)(OH)_2_(cis-1,4-DACH)] *(**1**)*

This compound was prepared according to a procedure reported in the literature with slight modifications [41]. Briefly, a solution of [Pt(CBDCA)(*cis*-1,4-DACH)] (100 mg; 0.22 mmol) in 40 mL of H_2_O was treated with a solution of H_2_O_2_ in H_2_O (30% *w*/*w*, 450 µL). The mixture was stirred at room temperature for 24 h in the dark. The resulting suspension was filtered and the filtrate was concentrated under reduced pressure to a minimum volume. Addition of acetone induced the formation of a white precipitate that was isolated by filtration of the mother liquor, washed with acetone, and dried under vacuum. Yield 77% (82 mg, 0.17 mmol). Anal.: calculated for C_12_H_22_N_2_O_6_Pt (**1**): C, 29.69; H, 4.57; N, 5.77%. Found: C, 29.53; H, 4.71; N, 5.63%. ESI-MS: calculated for C_12_H_22_N_2_O_6_PtNa [**1** + Na]^+^: 508.10. Found: 508.10 *m*/*z*. ^1^H-NMR (DMSO-d_6_): 6.47 (4H, NH_2_), 2.80 (2H, CH*a*), 2.52 (4H, CH*d*,*f*), 2.02 (4H, CH*b*), 1.77 (2H, CH*e*), 1.48 (4H, CH*c*), 0.35 (2H, OH) ppm. ^195^Pt NMR (DMSO-d_6_): 1617.60 ppm. ^13^C-NMR (DMSO-d_6_): 47.57 (C*a*), 31.67 (C*d*,*f*), 19.23 (C*b*,*c*), 15.69 (C*e*) ppm.

### 3.3. Synthesis of cis,trans,cis-[Pt(CBDCA)(ALA)_2_(cis-1,4-DACH)] (ALA = (±)-α-Lipoic Acid) *(**2**)*

α-Lipoic anhydride was prepared according to a procedure reported in the literature with slight modifications [42]. A mixture of (±)-α-lipoic acid (727 mg, 3.52 mmol) and DCC (494 mg, 2.39 mmol) was stirred in 26 mL of methylene chloride for about 15 h at room temperature. The solution was filtered, to remove the byproduct dicyclohexylurea (DCU), treated with 4-(dimethylamino)pyridine (1.4 mg, 0.0117 mmol) and **1** (57 mg, 0.117 mmol) and left under stirring at room temperature for 24 h. The resulting suspension was filtered and the solution treated with 120 mL of diethyl ether which induced the formation of a white precipitate that was isolated by filtration of the mother liquor, washed several times with diethyl ether, and dried under vacuum. Yield 35% (35 mg, 0.041 mmol). Anal.: calculated for C_28_H_46_N_2_O_8_PtS_4_·1/3diethyl ether (**2**·1/3 C_4_H_10_O): C, 39.73; H, 5.61; N, 3.25%. Found: C, 39.73; H, 5.45; N, 3.43%. ESI-MS: calculated for C_28_H_46_N_2_O_8_PtS_4_Na [**2** + Na]^+^: 885.16. Found: *m*/*z* 885.15. ^1^H-NMR (DMSO-d_6_): 7.76 (4H, NH_2_), 3.58 (2H, CH*ε*; see Scheme 3 for numbering of protons), 3.17 (2H, CH*η*’), 3.11 (2H, CH*η*), 2.84 (2H, CH*a*), 2.40 (4H, CH*d*,*f*), 2.39 (2H, CHζ’), 2.22 (4H, CHα), 1.85 (2H, CHζ), 1.78 (2H, CH*e*), 1.63 (8H, CH*b*,*c*), 1.62 (2H, CH*δ*’), 1.51 (2H, CH*δ*), 1.48 (4H, CH*β*), 1.32 (4H, CHγ) ppm. ^195^Pt NMR (DMSO-d_6_): 1932.70 ppm. ^13^C-NMR (DMSO-d_6_): 55.93 (Cε), 48.02 (C*a*), 39.69 (Cζ), 37.98 (C*η*), 35.69 (Cα), 33.92 (C*δ*), 30.47 (C*d*,*f*), 28.09 (Cγ), 24.73 (C*β*), 19.35 (C*b*,*c*), 15.21 (C*e*) ppm.

### 3.4. Synthesis of cis,trans,cis-[PtCl_2_(ALA)_2_(cis-1,4-DACH)] *(**3**)*

This compound was prepared as described for complex **2** with some differences in the purification step. Briefly, a solution of ALA (448 mg, 2.17 mmol) and DCC (299 mg, 1.45 mmol) was stirred in 16 mL of methylene chloride for about 15 h at room temperature. The byproduct DCU was removed by filtration and the solution treated with 4-(dimethylamino)pyridine (0.88 mg, 0.0072 mmol) and *cis*,*trans*,*cis*-[PtCl_2_(OH)_2_(*cis*-1,4-DACH)] (30 mg, 0.072 mmol) and left under stirring at room temperature for 24 h. The suspension was filtered and the resulting yellow solution was concentrated to 10 mL under reduced pressure. Addition of 40 mL of diethyl ether and 10 mL of *n*-pentane induced the formation of a yellow precipitate that was isolated by filtration of the mother liquor, washed several times with diethyl ether, and dried under vacuum. Yield 53% (30 mg, 0.038 mmol). Anal.: calculated for C_22_H_40_Cl_2_N_2_O_4_PtS_4_·1/3diethyl ether (**3**·1/3 C_4_H_10_O): C, 34.36; H, 5.36; N, 3.44%. *Found:* C, 34.56; H, 5.19; N, 3.90%. ESI-MS: calculated for C_22_H_40_Cl_2_N_2_O_4_PtS_4_Na [**3** + Na]^+^: 813.07. Found: *m*/*z* 813.07. ^1^H-NMR (DMSO-d_6_): 8.19 (4H, NH_2_), 3.59 (2H, CH*ε*; see Scheme 3 for numbering of protons), 3.23–3.08 (4H, CH*η*’,*η*), 2.98 (2H, CH*a*), 2.43 (2H, CHζ’), 2.26 (4H, CHα), 1.88 (2H, CHζ), 1.67 (2H, CH*δ*’), 1.61 (8H, CH*b*,*c*), 1.54 (2H, CH*δ*), 1.52 (4H, CH*β*), 1.38 (4H, CHγ) ppm. ^195^Pt NMR (DMSO-d_6_): 1217.60 ppm. ^13^C-NMR (DMSO-d_6_): 55.79 (C*ε*), 49.47 (C*a*), 39.62 (Cζ), 37.72 (C*η*), 35.93 (Cα), 33.92 (C*δ*), 27.93 (Cγ), 24.84 (C*β*), 19.76 (C*b*,*c*) ppm.

### 3.5. Experiments with Cultured Human Cells

Pt(IV) compounds **2** and **3** were dissolved in DMSO just before running the experiment and a calculated amount of drug solution was added to the cell growth medium to a final DMSO concentration of 0.5%, which had no detectable effect on cell viability. Cisplatin, kiteplatin, and [Pt(CBDCA)(*cis*-1,4-DACH)] were dissolved in 0.9% NaCl solution.

### 3.6. Cell Culture Studies

Human lung (H157), colon (HCT-15), and breast (MCF-7) carcinoma cell lines were obtained by American Type Culture Collection (ATCC, Rockville, MD, USA). The human ovarian 2008 adenocarcinoma cells were kindly provided by Prof. G. Marverti (Dept. of Biomedical Science of Modena University, Modena, Italy). Human cervical (A431) carcinoma cells were kindly provided by Prof. F. Zunino (Istituto Nazionale per lo Studio e la Cura dei Tumori, Milan, Italy). Cell lines were maintained using the following culture media containing 10% fetal calf serum (Euroclone, Milan, Italy), antibiotics (50 units/mL penicillin and 50 μg/mL streptomycin), and l-glutamine (2 mM): (i) RPMI for HCT-15, A431, MCF-7 and 2008 cells; (ii) DMEM medium for A375 cells.

### 3.7. Cytotoxicity Assays

The growth inhibitory effect toward tumor cell lines was evaluated by means of the MTT as previously described [24]. Cancer cells were seeded in 96-well microplates in growth medium (100 μL, 3–8 × 10^3^ cells/well, depending upon the growth characteristics of the cell line) and then incubated in a 5% carbon dioxide atmosphere at 37 °C. Following 24 h, the medium was replaced with a fresh one containing the compound to be tested. Triplicate cultures were established for each treatment. After 72 h, 10 μL of a 5 mg/mL MTT saline solution were added to each well and microplates were incubated for five additional hours. Subsequently 100 μL of a sodium dodecyl sulfate (SDS) solution in 0.01 M HCl were added to each well. After an overnight incubation, the inhibition of cell growth induced by the tested compound was evaluated by measuring the absorbance at 570 nm using a BioRad 680 microplate reader (BioRad Laboratories S.r.L.; Segrate, Italy). The average absorbance for each drug dose was expressed as a percentage of the control and plotted versus drug concentration. IC_50_ values were obtained from the dose-response curves by means of the 4-PL model (*p* < 0.05). IC_50_ values are the drug concentrations that reduce the mean absorbance at 570 nm to 50% of those of the untreated control wells.

### 3.8. Spheroid Cultures

Spheroids were initiated in liquid overlay by seeding 3 × 10^3^ A431 cells/well in phenol red free RPMI-1640 medium (Sigma Chemical Co.; Sigma-Aldrich, Milan, Italy), containing 10% FCS and supplemented with 20% methyl cellulose stock solution. A total of 150 μL of this cell suspension was transferred to each well of a round bottom, non-tissue culture 96 well-plate (Greiner Bio-one, Kremsmünster, Austria) to allow spheroid formation within 72 h.

### 3.9. ROS Production

The production of ROS was measured in A431 cells (10^4^ cells per well) grown for 24 h in 96-well plates in RPMI medium without phenol red (Sigma Chemical Co.). Cells were then washed with PBS and loaded with 10 μM 5-(and-6)-chloromethyl-2′,7′-dichlorodihydrofluorescein diacetate, acetyl ester (CM–H_2_DCFDA; Molecular Probes-Invitrogen) for 25 min, in the dark. Afterwards, cells were washed with PBS and incubated with increasing concentrations of tested complexes. Fluorescence increase was estimated with a plate reader (Fluoroskan Ascent FL, Labsystem, Finland) at 485 (excitation) and 527 nm (emission). Antimycin (1 μM, Sigma Chemical Co.), a potent inhibitor of Complex III in the electron transport chain, was used as positive control.

### 3.10. Mitochondrial Membrane Potential (ΔΨ)

The *ΔΨ* was assayed using the Mito-ID^®^ Membrane Potential Kit according to the manufacturer’s instructions (Enzo Life Sciences, Farmingdale, NY, USA). Briefly, A431 cells (5 × 10^3^ cells per well) were seeded in 96-well plates; after 24 h, cells were washed with PBS and loaded with Mito-ID Detection Reagent for 30 min at 37 °C in the dark. Afterwards, cells were washed with PBS and incubated with increasing concentrations of tested complexes. Fluorescence was estimated using a plate reader (Fluoroskan Ascent FL, ThermoScientific, Vantaa, Finland) at 490 nm (excitation) and 590 nm (emission).

### 3.11. Transmission Electron Microscopy (TEM) Analyses

About 10^6^ A431 cells were seeded in 24-well plates and, after 24 h incubation, treated with the tested compounds and incubated for additional 24 h. Cells were then washed with cold PBS, harvested, and directly fixed with 1.5% glutaraldehyde buffer with 0.2 M sodium cacodylate, pH 7.4. After washing with buffer and post-fixation with 1% OsO_4_ in 0.2 M cacodylate buffer, specimens were dehydrated and embedded in epoxy resin (Epon Araldite; Fisher Scientific Italia, Rodano (MI), Italy). Sagittal serial sections (1 μm) were counterstained with toluidine blue; thin sections (90 nm) were given contrast by staining with uranyl acetate and lead citrate. Micrographs were taken with a Hitachi H-600 electron microscope (Hitachi, Tokyo, Japan) operating at 75 kV. All photos were typeset in Corel Draw 11.

### 3.12. Statistical Analysis

All values are the means ± S.D. of no less than three measurements starting from three different cell cultures. Multiple comparisons were made by ANOVA followed by the Tukey–Kramer multiple comparison test (** *p* < 0.01) using GraphPad Prism 5.03 for Windows software (GraphPad Software, La Jolla, CA, USA).

## 4. Conclusions

A considerable amount of evidence has demonstrated that platinum drugs are activated intracellularly by aquation of the leaving groups and subsequent covalent binding to DNA, forming DNA adducts capable to activate various signal-transduction pathways such as those involved in DNA-damage recognition and repair, cell-cycle arrest, and programmed cell death or apoptosis. The Pt-DNA adducts cause unwinding and bending of double helix DNA that are recognized by many cellular proteins. Additional intracellular targets have been used in recent years to obtain more potent Pt complexes, mostly explored have been Pt(IV) complexes which allow the presence of two additional ligands in the axial positions suitable for the attachment of other cancer-targeting ligands [43,44]. In a previous work, this result was obtained by coordinating in the axial positions the ligand DCA, an orphan drug capable to inhibit the mitochondrial PDK [24]. Here we have extended this strategy by coordinating in the axial positions the antioxidant ALA. The new compounds (*cis*,*trans*,*cis*-[Pt(CBDCA)(ALA)_2_(*cis*-1,4-DACH)] (**2**) and *cis*,*trans*,*cis*-[PtCl_2_(ALA)_2_(*cis*-1,4-DACH)] (**3**)), after intracellular reduction, are capable to release kiteplatin (or its CBDCA analogue) and two molecules of α-Lipoic acid. The Pt(II) residue reaches its target DNA, while ALA could act on mitochondria as activator of the pyruvate dehydrogenase complex and could suppress anaerobic glycolysis.

Compounds **2** and **3** were prepared and thoroughly characterized by means of spectroscopic and spectrometric techniques and their in vitro cytotoxicity was tested on a panel of five human cancer cell lines and compared to that of cisplatin, oxaliplatin, and kiteplatin. Compounds **2** and **3** were much more effective than the reference compounds, with complex **3** proving to be the most effective also in 3D spheroid tumor cells. However, treatment of A431 cells with **2** and **3** did not determine an increase in cellular ROS basal production, usually correlated with the inhibition of mitochondrial PDK. In addition, treatment of A431 cells with **2** and **3** did not induce a significant depolarization of the mitochondrial membrane or any morphological alteration of mitochondria.

The overall results hence suggest that the potentiated activity of the Pt(IV) conjugates **2** and **3**, with respect to their Pt(II) precursors, can be due to other types of interactions promoted by the release of the ALA ligands (at micromolar concentration reached into the treated tumor cells), which, however, do not appear to significantly affect the macroscopic mitochondrial membrane potential and the mitochondrial morphological parameters. This aspect deserves further investigation. Moreover, since it has been shown that pretreatment with ALA significantly attenuates the effect of cisplatin on HEI-OC1 cells and prevents cisplatin-induced nephrotoxicity in rats, we are planning to evaluate the toxicity profiles by in vivo investigations.

## Figures and Tables

**Table 1:** In vitro antitumor activity.

Compound	IC_50_ (µM) ± S.D.					
	2008	MCF-7	A431	HCT-15	H157	Average
[Pt(CBDCA)(*cis*-1,4-DACH)]	25.47 ± 4.11	20.58 ± 3.15	13.56 ± 2.18	30.58 ± 5.25	19.21 ± 4.15	21.9
**2**	3.15 ± 0.85	3.05 ± 0.89	2.25 ± 0.52	2.31 ± 0.56	3.44 ± 0.97	2.8
**3**	0.34 ± 0.09	1.12 ± 0.57	0.10 ± 0.04	0.61 ± 0.15	0.99 ± 0.31	0.6
CDDP	2.22 ± 1.02	10.58 ± 0.82	2.10 ± 0.87	15.28 ± 2.63	2.12 ± 0.89	6.5
OXP	1.65 ± 0.46	4.52 ± 0.95	3.71 ± 0.76	1.15 ± 0.43	5.99 ± 1.85	3.4
Kiteplatin	1.89 ± 1.04	3.10 ± 1.42	3.95 ± 1.11	2.66 ± 0.95	2.08 ± 0.66	2.7

CDDP: cisplatin, OXP: oxaliplatin. S.D. = standard deviation. Cells (3–5 × 10^4^ mL^−1^) were treated for 72 h with different concentrations of tested compounds. Cytotoxicity was assessed by the MTT test. IC_50_ values were calculated by a four parameter logistic model (*p* < 0.05).

**Table 2:** Cytotoxicity towards human A431 cancer cell spheroids.

	IC_50_ (µM) ± S.D.					
	[Pt(CBDCA)(*cis*-1,4-DACH)]	2	3	Kiteplatin	CDDP	OXP
A431	98.9 ± 6.8	58.4 ± 3.8	30.2 ± 5.1	65.2 ± 5.8	71.1 ± 3.9	65.3 ± 5.2

Spheroids (3 × 10^3^ cells/well) were treated for 72 h with increasing concentrations of tested compounds. The growth inhibitory effect was evaluated by means of MTT test. IC_50_ values were calculated from the dose-survival curves by the four parameter logistic model (*p* < 0.05). S.D. = standard deviation.

## Supplementary Materials

Supplementary materials can be found at http://www.mdpi.com/1422-0067/19/7/2050/s1.

## Abbreviations

ALA	(±)-α-Lipoic acid
CBDCA	1,1-cyclobutanedicarboxylate
CCCP	Carbonylcyanide m-chlorophenyl hydrazone
CDDP	cisplatin
CM–H2DCFDA	5-(and-6)-chloromethyl-2′,7′-dichlorodihydrofluorescein diacetate, acetyl ester
COSY	correlation spectroscopy
DCA	dichloroacetate
DCC	dicyclohexylcarbodiimmide
DACH	diaminocyclohexane
DHALA	dihydro α-lipoic acid
DCU	dicyclohexylurea
DMEM	Dulbecco’s Modified Eagle Medium
DMSO	dimethylsulfoxide
ESI-MS	Electrospray Ionisation Mass Spectrometry
FCS	Fetal calf serum
HSQC	Heteronuclear single quantum coherence spectroscopy
MTT	3-(4,5-dimethylthiazol-2-yl)-2,5-diphenyltetrazolium bromide
OXP	oxaliplatin
PBS	phosphate buffer saline
ROS	reactive oxygen species
RPMI	Roswell Park Memorial Institute
SDS	sodium dodecyl sulfate
TEM	Transmission electron microscopy
