# Supplementary material for: Multi-Acting Mitochondria-Targeted Platinum(IV) Prodrugs of Kiteplatin with α-Lipoic Acid in the Axial Positions
Source: Int J Mol Sci. 2018 Jul 14;19(7):2050. doi: 10.3390/ijms19072050 (PMC6073472; doi:10.3390/ijms19072050)
Supplement: Supplementary file 1 [file ijms-19-02050-s001.pdf]

Supplementary Information

Multi-Acting Mitochondria-Targeted Platinum(IV) Prodrugs of Kiteplatin with α-Lipoic acid in the Axial Positions

Salvatore Savino,[1] Cristina Marzano,[2] Valentina Gandin,[2] James D. Hoeschele,[3] Giovanni Natile,[1,*] Nicola Margiotta.[1,*]

[1] Department of Chemistry, University of Bari Aldo Moro, Via E. Orabona 4, 70125, Bari (Italy);

[2] Department of Pharmaceutical and Pharmacological Sciences, University of Padua, Via Marzolo 5, 35131, Padova (Italy);

[3]Department of Chemistry, Eastern Michigan University, 48197 Ypsilanti, MI (USA).

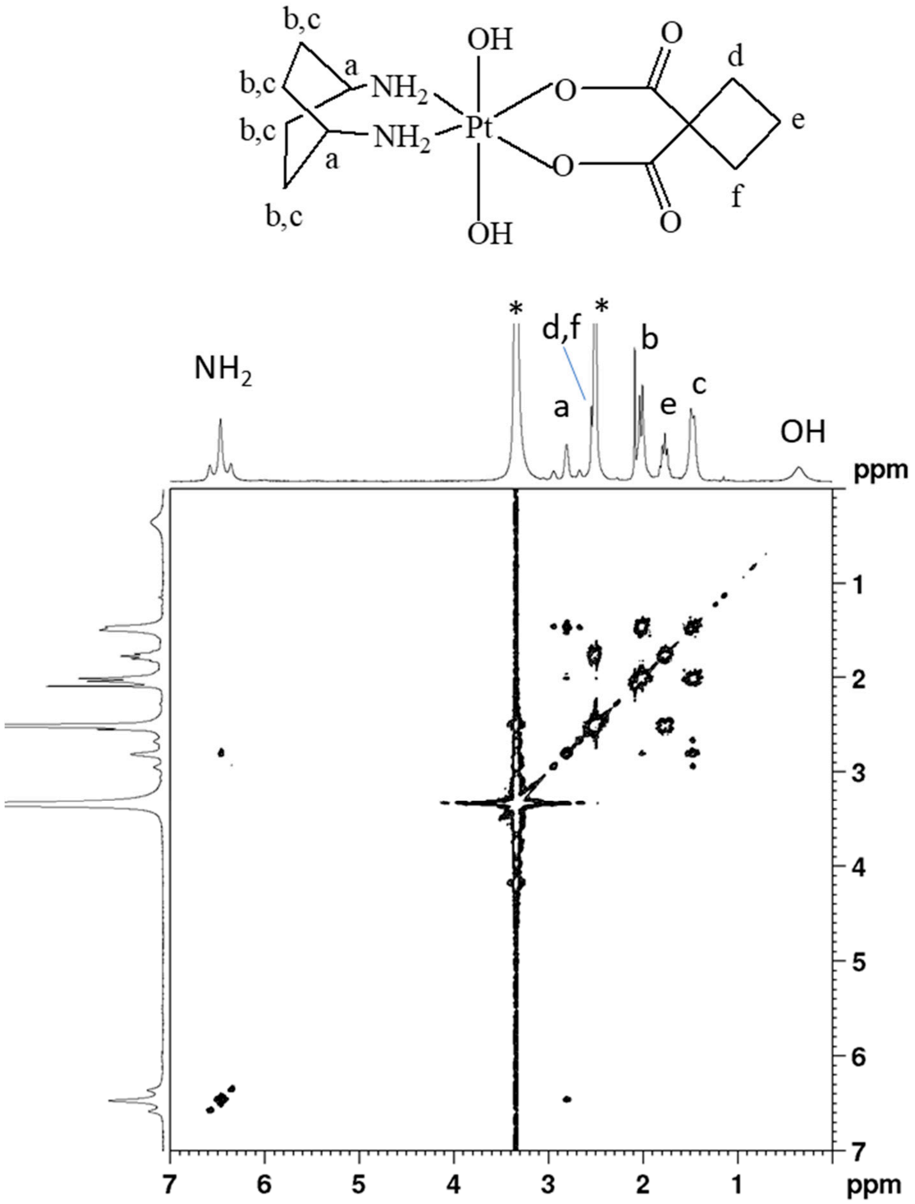

Figure S1. Selected region of the 2D COSY (700 MHz) spectrum obtained for *cis,trans,cis*-[Pt(CBDCA)(OH)$_2$(*cis*-1,4-DACH)] (**1**) in DMSO-d$_6$. The asterisks indicate the residual solvent peak.

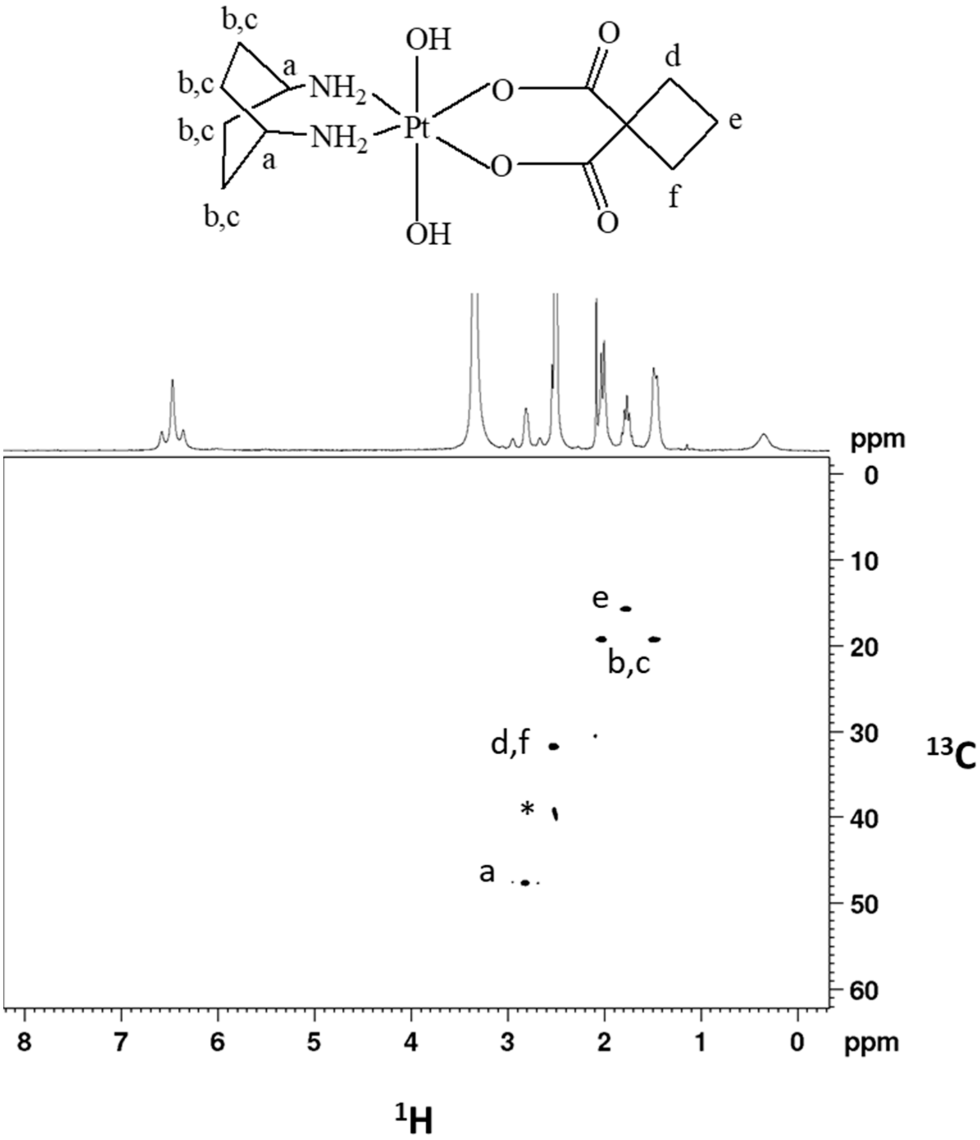

Figure S2. [^1H, ^{13}C] HSQC (^1H 700 MHz) of *cis,trans,cis*-[Pt(CBDCA)(OH)$_2$(*cis*-1,4-DACH)] (**1**) in DMSO-d$_6$. The asterisk indicates the residual solvent peak.

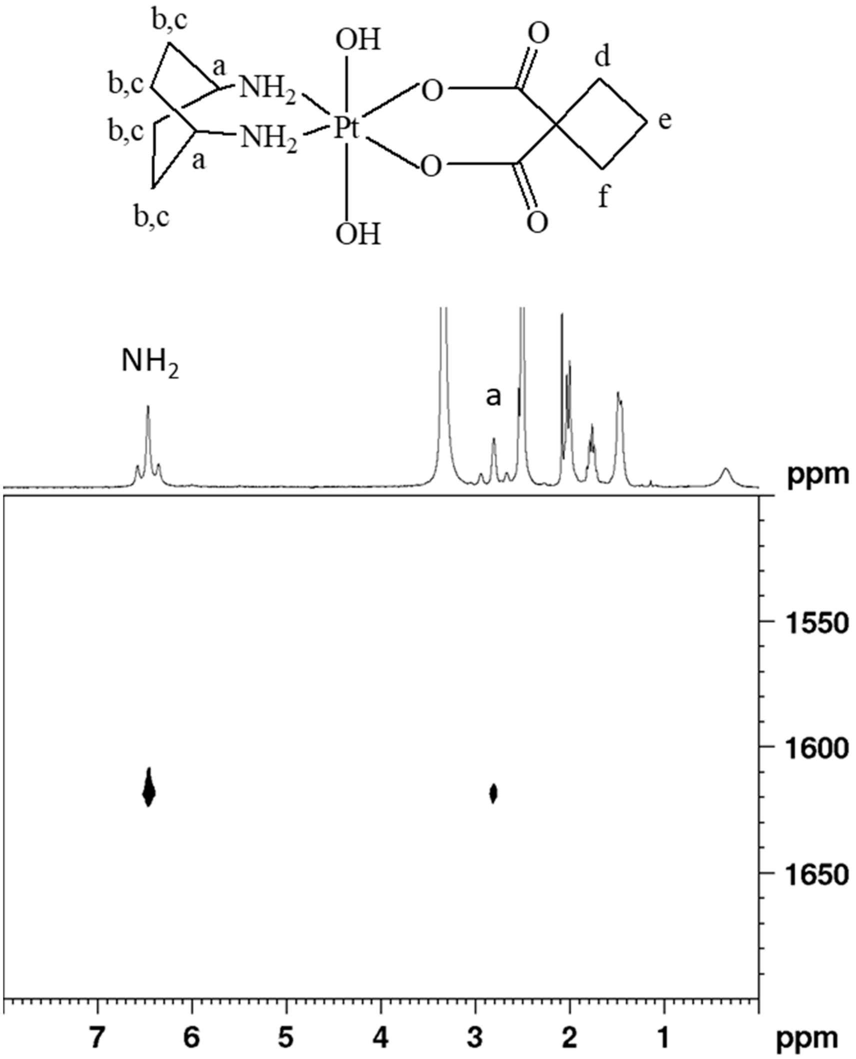

Figure S3. [^{1}H-^{195}Pt]-HSQC 2D spectrum (^{1}H 300 MHz) obtained for *cis,trans,cis*-[Pt(CBDCA)(OH)$_2$(*cis*-1,4-DACH)] (**1**) in DMSO-d$_6$.

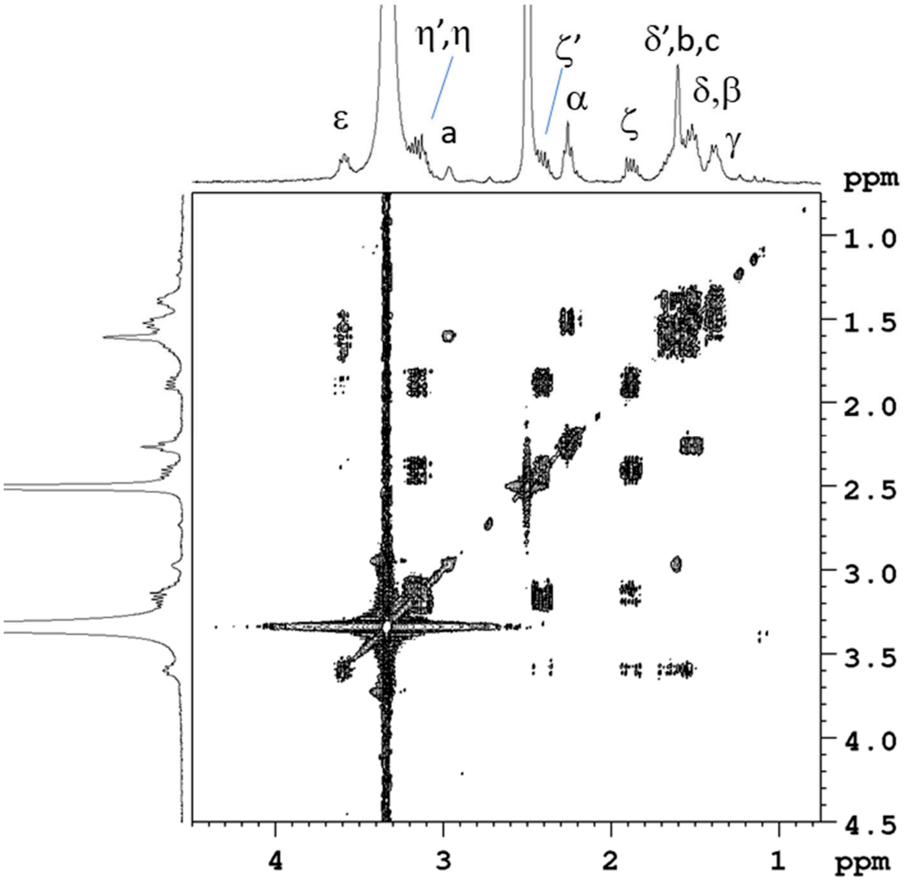

Figure S4. Selected region of the 2D COSY (700 MHz) spectrum obtained for *cis,trans,cis*-[$PtCl_2(ALA)_2$(*cis*-1,4-DACH)] (**3**) in DMSO-d$_6$. Numbering of protons is reported in Scheme 3.

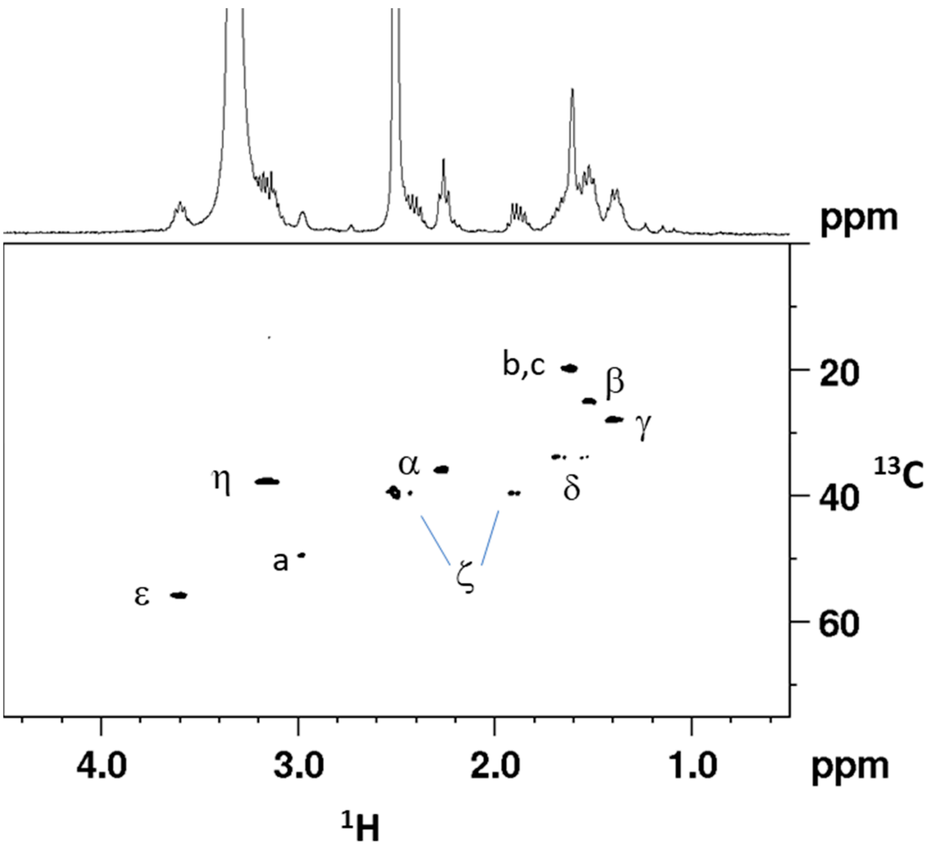

Figure S5. [^{1}H, ^{13}C] HSQC (^{1}H 700 MHz) of *cis,trans,cis*-[$PtCl_2(ALA)_2$(*cis*-1,4-DACH)] (**3**) in DMSO-d_6. Numbering of protons is reported in Scheme 3.

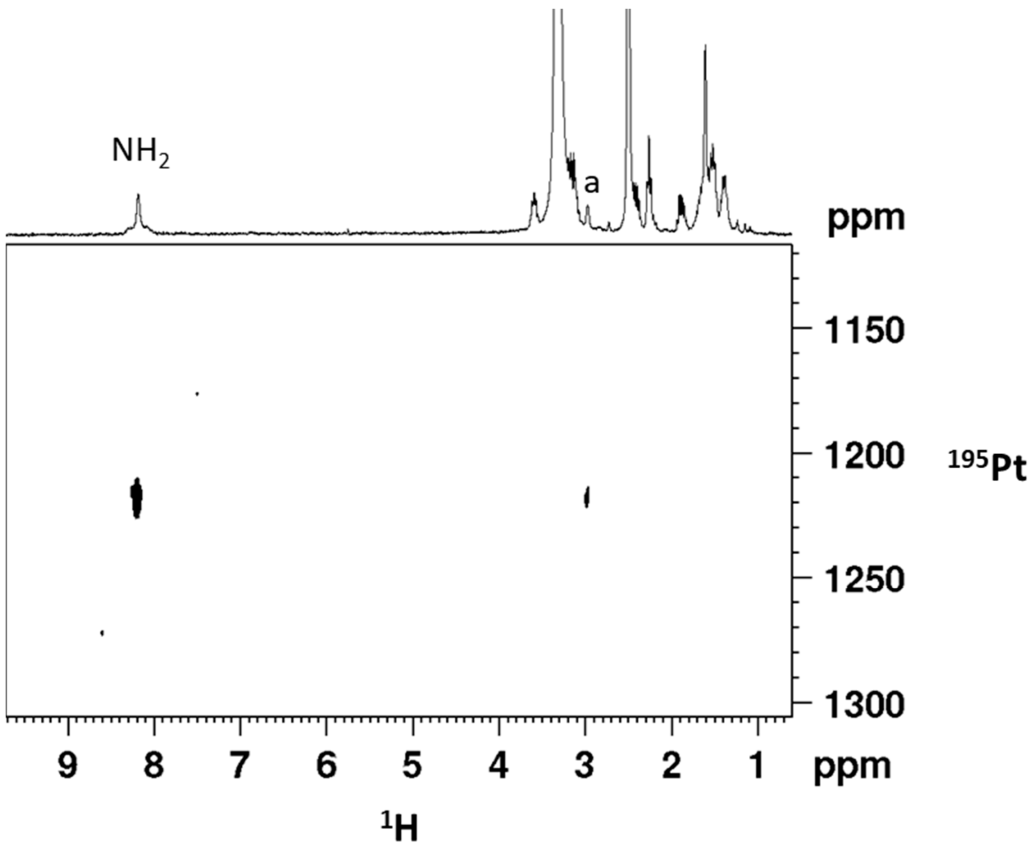

Figure S6. [^{1}H-^{195}Pt]-HSQC 2D spectrum (^{1}H 300 MHz) obtained for *cis,trans,cis*-[$PtCl_2(ALA)_2$(*cis*-1,4-DACH)] (**3**) in DMSO-d_6. Numbering of protons is reported in Scheme 3.
